# Peripheral and ocular microvascular alterations in systemic sclerosis: observations from capillaroscopic assessments, perfusion peripheral analysis, and optical coherence tomography angiography

**DOI:** 10.1007/s00296-023-05495-z

**Published:** 2023-11-17

**Authors:** Carlo Alberto Cutolo, Andrea Cere, Paola Toma, Tommaso Cannavacciuolo, Chiara Toma, Serena Balito, Veronica Gerli, Vanessa Smith, Alberto Sulli, Sabrina Paolino, Emanuele Gotelli, Carlo Enrico Traverso, Massimo Nicolò, Maurizio Cutolo, Elvis Hysa

**Affiliations:** 1 Ophthalmology Clinic DiNOGMI, University of Genova, Genoa, Italy; 2 Laboratory of Experimental Rheumatology and Academic Division of Clinical Rheumatology, Department of Internal Medicine, University of Genova, Genoa, Italy; 3 IRCCS San Martino Polyclinic Hospital, Genoa, Italy; 4 Department of Internal Medicine, Ghent University, Ghent, Belgium; 5 Department of Rheumatology, Ghent University Hospital, Corneel Heymanslaan 10, Ghent, Belgium; 6 Unit for Molecular Immunology and Inflammation, VIB Inflammation Research Center (IRC), Corneel Heymanslaan 10, 9000 Ghent, Belgium

**Keywords:** Systemic sclerosis, Capillaroscopy, Laser doppler flowmetry, Optical coherence, Tomography, Diagnostic imaging

## Abstract

To investigate the correlations between finger microvascular morphology and function in patients with systemic sclerosis (SSc) and the status of ocular microcirculation, as detected by nailfold videocapillaroscopy (NVC), laser speckle contrast analysis (LASCA), and optical coherence tomography angiography (OCTA). The enrollment included 32 SSc patients, classified according to the 2013 ACR/EULAR criteria, and 27 sex- and age-matched healthy controls. The participants underwent comprehensive rheumatological and ophthalmological examinations, as well as NVC, LASCA, and OCTA analysis on the same day at a single center from March to October 2022. SSc patients receiving intravenous prostanoids cycles were assessed at least 1 month after infusion. Statistical analysis was conducted using Stata® 15.1. Significant direct correlations were observed between the mean capillary number (at NVC) and the mean perfusion of fingers (at LASCA) with the retinal and choroidal perfusion (at OCTA) (all *p* < 0.05). In addition, a significantly reduced retinal and choroidal perfusion was detected in SSc patients vs controls (all *p* < 0.05). Interestingly, diffuse cutaneous SSc (dcSSc) patients exhibited a lower choroidal perfusion (*p* = 0.03) but an increased choroidal thickness (CT) than limited cutaneous SSc patients (*p* < 0.001). CT was increased also in patients with positive Scl70 antibodies and with a history of digital ulcers directly correlating with disease duration (*r* = 0.67, *p* = 0.001). Finally, the combination of LASCA and OCTA parameters showed a significant discrimination capacity between SSc patients and controls, with an area under the curve of 0.80 [95% CI (0.74, 0.87)]. Peripheral microvascular damage is correlated with impaired ocular microcirculation in SSc. The increased choroidal thickness observed in dcSSc may be related to local sub-endothelial extracellular matrix deposition. The combined analysis of choroidal and fingertip perfusion offers preliminary insights that may complement traditional diagnostic methods for SSc.

**Supplementary Information:**

The online version contains supplementary material available at 10.1007/s00296-023-05495-z.

## Introduction

Systemic sclerosis (SSc) is a rare and complex autoimmune disease, occurring more frequently in women and featured by microvascular damage, aberrant activation of innate and adaptive immunity with production of specific autoantibodies together with progressive fibrosis of skin and visceral organs [1].

A high grade of inter-patient variability characterizes SSc with multiple clinical phenotypes, beginning from the extent of the skin involvement to the autoantibody profile, disease progression, and organ damage severity (lung, kidneys, heart, and gastrointestinal tract) which heavily conditions response to treatment and overall survival [2].

Considering the rarity of the disease, SSc-related ocular manifestations have been mainly reported in case reports and small case series [3, 4]. A recent systematic literature review investigating ocular changes in SSc has reported that the most common eye disorders are eyelid skin fibrosis, dry eye disease, and involvement of the posterior segment with a prominent involvement of the choroidal microvasculature compared with the retinal microcirculation, investigated by optical coherence tomography angiography (OCTA) [5]. Conversely, some recent studies have also shown that early retinal microvascular alterations in patients with SSc, particularly a reduction of the vessel density, occur without any clinical evidence of retinopathy suggesting the hypothesis of a widespread vascular injury [6–8].

In addition, few studies with contrasting results have assessed, in SSc patients, correlations between retinal findings and the parameters of nailfold videocapillaroscopy (NVC) which can easily identify the specific qualitative and quantitative alterations of the peripheral microvascular status induced by the disease progression [9–13].

However, there are no published reports, to our knowledge, which have investigated the correlations between the functional peripheral finger perfusion of SSc patients, evaluated with techniques of dynamic imaging, and the ocular microvascular status.

In this cross-sectional monocentric study, our primary endpoint was to correlate, both peripheral morphological microvascular statuses assessed by NVC and functional perfusion analyzed by laser speckle contrast analysis (LASCA) with the OCTA-derived data among the SSc cohort.

Second, we aimed to compare OCTA variables observed in SSc patients with age- and sex- matched healthy controls (HCs). Additional secondary endpoints investigated the correlations between OCTA variables and clinical features of SSc patients.

Last, we assessed the degree of the performance of OCTA and LASCA as classifiers for distinguishing SSc patients from HCs and for differentiating subtypes of SSc patients. The rationale of the choice of these imaging techniques without NVC derives from the already known high discriminative capacity of NVC in distinguishing SSc patients from HCs through the recognition of the scleroderma patterns [14, 15].

This endeavor was driven by our objective to offer an integrated perspective on the peripheral and ocular vascular alterations of SSc providing insights into the connections between these microcirculatory districts.

## Methods

### Study population

A population of 33 consecutive SSc patients followed-up at Rheumatology Division of Genova University (Italy) and 30 age- and sex-matched HCs were analyzed between March 2022 and October 2022 after providing the written informed consent provided by the University Hospital (CONSAZHQA_0001). SSc patients were classified during the standard visits according to 2013 ACR/EULAR criteria [16]. This study was performed according to the principles of Good Clinical Practices and the Declaration of Helsinki following the local Ethical Board Committee approval (protocol ID: 237REG2015).

All participants underwent a baseline ophthalmological examination including ocular biometry and OCTA and a peripheral microvascular assessment by NVC and LASCA.

Our exclusion criteria were the following: underage patients (< 18 years old), active smokers, underlying malignancies, systemic untreated infections (i.e., HBV, HCV, and HIV), and clinical conditions which could represent a bias for microvascular assessment at OCTA, NVC, and LASCA, such as diabetes, severe uncontrolled systemic hypertension, peripheral atherosclerotic disease, coexisting ocular diseases (including refraction anomalies such as myopia, cataracts, glaucoma or a previous diagnosis of retinopathy), and optical media opacities that could impair the quality of the OCT scans. Uneventful cataract surgery or laser capsulotomy performed more than 6 months before the present study was not considered an exclusion criterion. This decision was taken considering the concepts of stable visual outcomes and clinical relevance. By choosing a 6-month interval, we ensured that patients had passed through this postoperative phase of these surgical procedures, allowing their visual status to reach a stable baseline, minimizing the potential for variability in visual parameters. In addition, the inclusion of this broader population of SSc individuals might enhance the generalizability of our findings and their relevance to real-world clinical scenarios.

### Nailfold videocapillaroscopy (NVC)

Nailfold videocapillaroscopy (NVC) was performed using an optical probe, equipped with a 200 × contact lens connected to image analysis software (Adamo S.r.l—Horus—Trapani, Italy). The same physician performed NVC examinations for the enrolled participants (EH) according to the standardized procedures [17]. According to recent consensus-based definitions, the NVC parameters were defined as following: normal capillaries (hairpin-shaped), non-specific capillary variations of morphology (tortuous or crossing capillaries with branch diameters < 20 μm), dilated capillaries (irregular or homogeneous increase of capillary diameter between 20 and 50 μm), giant capillaries (homogeneously dilated normal shaped loops with a diameter ≥ 50 μm), microhemorrhages (dark lesions attributable to hemosiderin deposit), abnormal shapes (i.e., ramified capillaries, non-convex head of capillaries, neoangiogenesis, originating from a single capillary), and lower capillary density (reduction of capillary number below normal range of 7 capillaries per linear mm, counted at distal row) [14, 17]. NVC parameters were collected, for all participants, on the same day of OCTA and LASCA exams. A semiquantitative rating scale was utilized to score microvascular findings (score 0–3) and the appropriate NVC pattern of microangiopathy (“early”, “active” or “late”) was assigned to SSc patients [18].

### Laser speckle contrast analysis (LASCA)

Peripheral blood perfusion (PBP) was analyzed by LASCA (laser speckle contrast analysis—Pericam PSI, Perimed, Jarfalla), in both HCs and SSc patients [19, 20]. SSc patients were not under the influence of prostanoid intravenous therapy for at least 4 weeks before the registration. After 15 min of acclimatization in the room at a temperature maintained between 20 and 25 °C, PBP was registered on the whole hands, both on the volar and palmar sides, for a total of 4 records of 30 s each one. Setting was set up to perform five images each second and the software automatically measured PBP as perfusion units (P.U.). Subsequently, regions of interest (ROIs) were created as follows: fingertips, periungual areas, palmar and volar aspect of the 2nd–3rd–4th–5th fingers, whole hands. The average PBP values between fingertips, periungual areas, and between right and left hands were measured.

### Rheumatologic assessment of SSc patients

All SSc patients underwent clinical evaluation, laboratory and instrumental exams as part of the regular follow-up [21], in a time frame not exceeding the 3 months before or after the performance of OCTA, NVC, and LASCA. Among clinical parameters, we assessed the skin involvement using the modified Rodnan skin score (mRSS; range 0–51) to measure cutaneous thickness and recorded dichotomously (yes/no) the presence of sclerodactyly, puffy fingers, telangiectasias, calcinosis, and digital ulcers. According to the extent of the skin involvement, SSc patients were classified as limited cutaneous (lc) or diffuse cutaneous (dc): more specifically patients were classified as dcSSc if the skin of the arms, trunk, and thighs were involved [22].

The visceral involvement of SSc patients was recorded dichotomously based upon the presence of interstitial lung disease (ILD) at HRTC for defining lung involvement, increased pulmonary arterial hypertension (PAP) at echocardiography and/or at right heart catheterization, performed when indicated by guidelines [23], for defining patients with pulmonary arterial hypertension (PAH), manometric evidence of esophageal dysmotility for defining esophageal involvement [24] and increased RRI/elevated serum creatinine levels for defining renal compromission.

Current treatment with immunosuppressive and/or vasoactive drugs and comorbidities were collected as well.

Antinuclear antibodies (ANA) were assessed using indirect immunofluorescence on Hep-2/liver cells (EUROPLUS ANA Mosaic FA 1510–1), with a 1: 80 serum dilution as cutoff value. SSc-related autoantibodies (SSc-Abs) were detected by ELISA (EUROASSAY Anti-ENA ProfilePlus 1 ELISA IgG, EA 1590-1G) and immunoblot (EUROLINE Systemic Sclerosis Profile (Nucleoli) DL 1532 G). More specifically, antibodies directed toward Scl-70, centromere A and centromere B, RNA polymerase III (RNAP-III), specifically RNAP-III 11 kDa (RP11) and RNAP-III 155 kDa (RP155), Ro-52, PM-Scl75, PM-Scl100, Ku, fibrillarin, Nor90, Th/To, and platelet-derived growth factor receptors (PDGF-R) were assessed [25]. Tests were performed according to the manufacturer’s instructions (EUROIMMUN AG, Lübeck, Germany).

### Ophthalmological assessment

All patients and HCs underwent a comprehensive ophthalmological exam which included best corrected visual acuity (BCVA) measured using the Snellen chart, slit lamp biomicroscopy of the anterior segment, and fundoscopy. Both eyes were examined in all subjects. Ocular biometry was achieved using the OA-2000 Optical Biometer (Tomey) to measure axial length (AL), anterior chamber depth (ACD), lens thickness (LT), keratometry, and central corneal thickness (CCT). Intraocular pressure (IOP) was assessed with Goldmann applanation tonometry.

### Optical coherence tomography angiography

Imaging was performed without prior pupil dilatation using swept-source (SS)-OCT and SS-OCTA (DRI OCT Triton; Topcon Corporation). OCT scans were obtained by a single trained examiner (CAC). All images were captured at rest and at the same time around 2 p.m. to avoid physiological variations of ocular perfusion.

Each imaging session included 3D-Wide scan and OCTA (4.5 × 4.5 mm) centered on the fovea. Only images of high quality, without motion, segmentation errors, or projection artifacts were accepted to guarantee reliable analysis.

The segmentation of retinal angiograms was performed using the built-in software of the SS-OCT, specifically the Topcon ImageNet 6. This software is designed to provide detailed en face images of the retinal layers, including the superficial capillary plexus (SCP), the deep capillary plexus (DCP), and the choriocapillaris slab (CC).

The Topcon ImageNet 6 software employs proprietary algorithms to segment these layers based on their optical properties and depth within the retina. The default settings provided by the manufacturer were utilized to produce en face images of the SCP between the inner limiting membrane (ILM) and the inner plexiform layer/inner nuclear layer (IPL/INL) counted between zero µm and 15.6 µm deep, the DCP between 15.6 µm and 70.2 µm, and the choriocapillaris slab (CC) estimated from the Bruch’s membrane (BM) up to 20.8 µm below BM.

One of the key features of the Topcon ImageNet 6 software is its ability to automatically segment these layers while allowing for manual adjustments when necessary. This ensures that the segmented images accurately represent the true anatomical structures of the retina.

### Interpretation of the imaging data

In the execution of this study, the imaging assessments were conducted by physicians trained in the respective imaging modalities (EH for NVC, AC for LASCA and CAC for OCTA and ocular variables). Subsequent interpretation and analysis of the acquired imaging data were carried out by the same physicians to ensure consistency and accuracy in the evaluation process. This approach was adopted to minimize potential biases and discrepancies that might have arisen from multiple interpreters.

### Statistical analysis

Data from the enrolled SSc patients and HCs who met the inclusion criteria were analyzed. The measurements from both eyes were averaged. In cases where one eye did not meet the criteria, only the data from the other eye were considered for analysis. This decision was based only for certain ocular conditions that are localized or monolateral, with the assumption that they affect only one eye and do not have a systemic or bilateral presentation. Examples of such conditions include monocular trauma, unilateral cataract development due to injury, localized ocular infections, or isolated branch retinal vein occlusion.

Continuous variables were summarized as medians and interquartile range (IQR) or mean and standard deviation (SD) when appropriate, while categorical variables were expressed as numbers and proportions. The distribution of data was assessed by the Kolmogorov–Smirnov test. To determine the significance of differences between healthy subjects and those with SSc, *t* tests, Wilcoxon tests, or Chi-squared tests were used based on the type and distribution of the data being analyzed. The correlation coefficient between meaningful NVC, LASCA, and ocular measurements was calculated through Pearson’s test and the Bonferroni corrected significance level was calculated.

The association between the diagnosis of SSc with ocular or LASCA data was analyzed by logistic regression. Different models were built including OCTA data, LASCA data, or both. These models were adjusted for meaningful confounding factors. Predictive performance was compared between models using area under receiver operating characteristic curves (AUC). Sensitivity and specificity values were reported at the cutoff points determined by the Youden index.

Any *p* values lower than 0.05 were considered statistically significant. The analysis was conducted using Stata 15.1 (StataCorp LLC, College Station, TX).

## Results

Data from 32 SSc patients (mean age 61.1 ± 13.4 years, mean disease duration 9.5 ± 6 years) and 27 HCs were analyzed. Four subjects were excluded from the analysis because of coexisting ocular conditions. Namely, three individuals in the control group suffered from age-related maculopathy whereas one SSc patient suffered from glaucoma. Age was positively correlated with the duration of the disease (*r* = 0.47, *p* < 0.01).

Table 1 summarizes the demographic, microvascular, and ocular characteristics of SSc compared with HCs.

**Table 1:** Demographic, peripheral microvascular and ophthalmological characteristics in SSc and HCs

Variable	SSc group (*n* = 32)	Control group (*n* = 27)	*p* value
Age, years	61.1 ± 13.4	58.0 ± 11.9	0.36
Gender:			0.33
Female	27 (84.4%)	25 (92.6%)	
Male	5 (15.6%)	2 (7.4%)	
Number of capillaries per linear mm at NVC	5.3 ± 1.4	9.4 ± 0.8	** < 0.01**
Fingertips perfusion, PU	143 ± 61	202 ± 60	** < 0.01**
Fingertips perfusion (thumbs excluded), PU	143 ± 60	203 ± 62	** < 0.01**
Periungual perfusion, PU	93 ± 48	145 ± 44	** < 0.01**
Periungual perfusion (thumbs excluded), PU	90 ± 47	144 ± 46	** < 0.01**
Spheral equivalent, diopters	− 0.24 ± 1.7	− 0.02 ± 2.0	0.65
LogMAR	0.03 ± 0.02	0.01 ± 0.02	0.11
Lens status			0.18
Phakic	29 (93.5%)	27 (100%)	
Pseudophakic	2 (6.5%)**	0 (0%)	
Axial length, mm	24.0 ± 1.5	23.7 ± 1.2	0.33
Anterior chamber depth, mm	3.33 ± 0.54	3.19 ± 0.33	0.25
Lens thickness, mm	4.45 ± 0.46	4.43 ± 0.49	0.84
Mean keratometry, diopters	43.8 ± 1.4	43.5 ± 1.9	0.39
Central corneal thickness, µm	533 ± 25	530 ± 49	0.476
IOP, mmHg	13.6 ± 2.3	11.9 ± 2.2	**0.006**

Significant *p*-values are in bold

Data are reported as means ± standard deviation or frequencies (%)

*SSc* systemic sclerosis, *HCs* healthy controls, *NVC* nailfold videocapillaroscopy, *PU* perfusion unit, *LogMAR* logarithm of the minimum angle of resolution, *IOP* intraocular pressure. Measures are average between the two eyes or average between the two hands. **Pseudophakia is bilateral

The two groups were similar in age, gender, visual ability, refractive status, and ocular biometric information, with no significant differences observed.

A correlation matrix showing the associations between peripheral microvascular variables and OCTA parameters of SSc patients is shown in Table 2. It was noteworthy the significant associations between NVC, LASCA, and OCTA parameters.

**Table 2:** Correlation matrix

	Age	AL	IOP	SCP	DCP	CC	CT	RT	RNFL	LASCA	NVC
Age	1										
AL	0.11 1.00										
IOP	0.06 1.00	0.09 1.00									
SCP	− 0.04 1.00	− 0.01 1.00	− 0.09 1.00								
DCP	− 0.17 1.00	− 0.01 1.00	− 0.16 1.00	**0.78** ** < 0.01**							
CC	− 0.18 0.78	0.05 1.00	− 0.15 1.00	0.14 1.00	**0.26** **0.03**						
CT	− **0.45** ** < 0.01**	− 0.47* ** < 0.01**	− 0.06 1.00	0.12 1.00	0.10 1.00	− 0.06 1.00					
RT	− 0.20 0.47	− 0.66* ** < 0.01**	− 0.05 1.00	− 0.05 1.00	− 0.09 1.00	0.08 1.00	**0.43** ** < 0.01**				
RNFL	− **0.27** **0.03**	− 0.58* ** < 0.01**	0.02 1.00	0.05 1.00	0.09 1.00	0.05 1.00	**0.45** ** < 0.01**	**0.57** ** < 0.01**			
LASCA	− 0.01 1.00	− 0.09 1.00	− **0.36** ** < 0.01**	0.12 1.00	**0.29** **0.01**	**0.28** **0.01**	− 0.08 1.00	0.06 1.00	0.24 0.08		
NVC	− 0.05 1.00	− 0.14 1.00	− **0.30** ** < 0.01**	**0.29** **0.01**	**0.25** **0.05**	0.23 0.10	0.12 1.00	0.22 0.19	0.15 1.00	**0.29** ** < 0.01**	
Disease duration	**0.47** ** < 0.01**	0.26 0.75	0.14 1.00	− 0.14 1.00	− 0.10 1.00	− 0.20 1.00	− **0.41** **0.01**	**0.57** ** < 0.01**			

Significant *p*-values are in bold

In each cell, the first line is the Pearson correlation coefficient, and the second line is the Bonferroni adjusted *p* value. *LASCA parameter* average of fingertip 2–5 P.U., *NVC parameter* mean n° of capillaries per linear mm, *AL* axial length, *IOP* intraocular pressure, *SCP* superficial capillary plexus, *DCP* deep capillary plexus, *CC* choriocapillaris, *CT* choroidal thickness, *RT* retinal thickness, *RNFL* retinal nerve fiber layer

*The inverse significant correlation between age and axial length (AL) with CT, RT, and RNFL is expected from an ophthalmological point of view

Indeed, the mean capillary number at NVC directly correlated with the retinal perfusion values of the SVP and DVP at OCTA (*r* = 0.3, *p* = 0.01 and *r* = 0.28, *p* = 0.01) and the mean peripheral perfusion detected by LASCA positively correlated with the DVP and CC perfusion (*r* = 0.29 and *r* = 0.28, both *p* = 0.01).

In addition, the mean number of capillaries per linear mm at NVC and the mean peripheral perfusion at LASCA from the 2nd to 5th fingertip were inversely correlated with the IOP (*r* = − 0.30 and *r* = − 0.36, both *p* < 0.01).

A significant difference emerged between SSc patients and HCs in terms of intraocular pressure (IOP): more specifically, IOP was found higher in SSc than in HCs (*p* = 0.006). Interestingly, no difference was detected in the thickness of the retinal nerve fiber layer (RNFL) between SSc patients and controls (Table 3). As expected, SSc patients showed significant differences from HCs in all NVC and LASCA variables (*p* < 0.01).

**Table 3:** Comparison of OCT- and OCTA-derived data between SSc and HCs

Variable	SSc (*n* = 32)	HCs (*n* = 27)	*p *value
Scan quality	61 ± 6	63 ± 5	0.315
Superficial capillary plexus, %	39.4 ± 2.4	40.8 ± 2.7	**0.040**
Deep capillary plexus, %	39.6 ± 2.7	41.3 ± 3.0	**0.026**
Choriocapillaris, %	51.1 ± 2.7	52.9 ± 1.5	**0.003**
Choroidal thickness, µm	212 ± 90	220 ± 68	0.725
Retinal thickness, µm	267 ± 28	275 ± 15	0.181
RNFL, µm	101 ± 11	102 ± 10	0.707

Significant *p*-values are in bold

*SSc* systemic sclerosis, *HCs* healthy controls, *RNFL* retinal nerve fiber layer

In Table 4, lcSSc and dcSSc patients are compared in terms of clinical and ocular features. No significant differences were detected between the two subgroups in the clinical domains except for the mean mRSS (*p* = 0.03) and a trend for dcSSc showing higher frequencies of ILD and previous digital ulcers. Concerning the autoantibody profile, lcSSc patients presented a significantly higher positivity for anti-centromere antibodies than dcSSc, as expected.

**Table 4:** Clinical features and ocular characteristics in lcSSc vs dcSSc

Variable	lcSSc, *n* = 22	dcSSc, *n* = 10	*p* value
Age (years)	63.5 ± 10.7	56.3 ± 18.2	0.185
Duration of the disease (years)	10.7 ± 6.0	8.5 ± 5.1	0.370
mRSS	5 ± 2	11 ± 5	**0.03**
Patients in treatment with oral vasodilators^1^ (%)	16 (72%)	6 (60%)	0.75
Patients in treatment with aspirin (%)	20 (90%)	6 (60%)	0.11
Patients in treatment with DMARDs^2^ (%)	20 (90%)	9 (90%)	0.99
Patients in treatment with nintedanib (%)	4 (18%)	1 (10%)	0.94
Scl-70 (%)	5 (20%)	5 (50%)	0.076
ACA (%)	13 (52%)	1 (10%)	**0.022**
Pulmonary involvement (%)	12 (48%)	8 (80%)	0.083
Pulmonary arterial hypertension (%)	3 (14%)	2 (20%)	0.21
Gastro-esophageal involvement (%)	8 (32%)	6 (60%)	0.130
Kidney involvement (%)	7 (28%)	4 (40%)	0.489
Previous or active digital ulcers (%)	7 (28%)	6 (60%)	0.076
Spheral equivalent, diopters	− 0.53 ± 1.49	0.68 ± 2.11	0.089
LogMAR	0.04 ± 0.10	0.01 ± 0.02	0.319
Axial length, mm	24.25 ± 1.60	23.29 ± 1.06	0.130
Anterior chamber depth, mm	3.44 ± 0.61	3.05 ± 0.29	0.104
Lens thickness, mm	4.43 ± 0.45	4.62 ± 0.48	0.330
Mean keratometry, diopters	44.1 ± 1.3	43.4 ± 1.4	0.230
Central corneal thickness, µm	537 ± 24	513 ± 17	0.09
IOP, mmHg	13.5 ± 2.5	13.9 ± 1.9	0.694
Scan Quality	60.6	60.7	0.982
Superficial vascular plexus, %	39.2 ± 2.7	39.4 ± 1.2	0.878
Deep vascular plexus, %	39.3 ± 3.0	39.6 ± 1.7	0.779
Choriocapillaris, %	51.8 ± 1.5	49.8 ± 3.6	**0.033**
Choroidal thickness, µm	176 ± 79	304 ± 57	** < 0.001**
Retinal thickness, µm	261 ± 32	278 ± 9	0.161
RNFL, µm	98 ± 12	107 ± 8	0.06

Significant *p*-values are in bold

*lcSSc* limited cutaneous SSc, *dcSSc* diffuse cutaneous SSc, *Scl-70* anti-topoisomerase I antibodies, *ACA* anti-centromere antibodies, *LogMAR* logarithm of the minimum angle of resolution, *IOP* intraocular pressure, *RNFL *retinal nerve fiber layer

^1^The vasodilators prescribed in the whole cohort included aminaphtone (69%), calcium channel blockers (19%), endothelin receptors inhibitors (32%), phosphodiesterase inhibitors (13%) or oral agonists of prostacyclin receptor (3%)

^2^The prescribed DMARDs among all SSc patients included hydroxychloroquine (13%), methotrexate (22%), mycophenolate mofetil (34%), rituximab (9%), cyclophosphamide (16%)

**Fig. 1:**
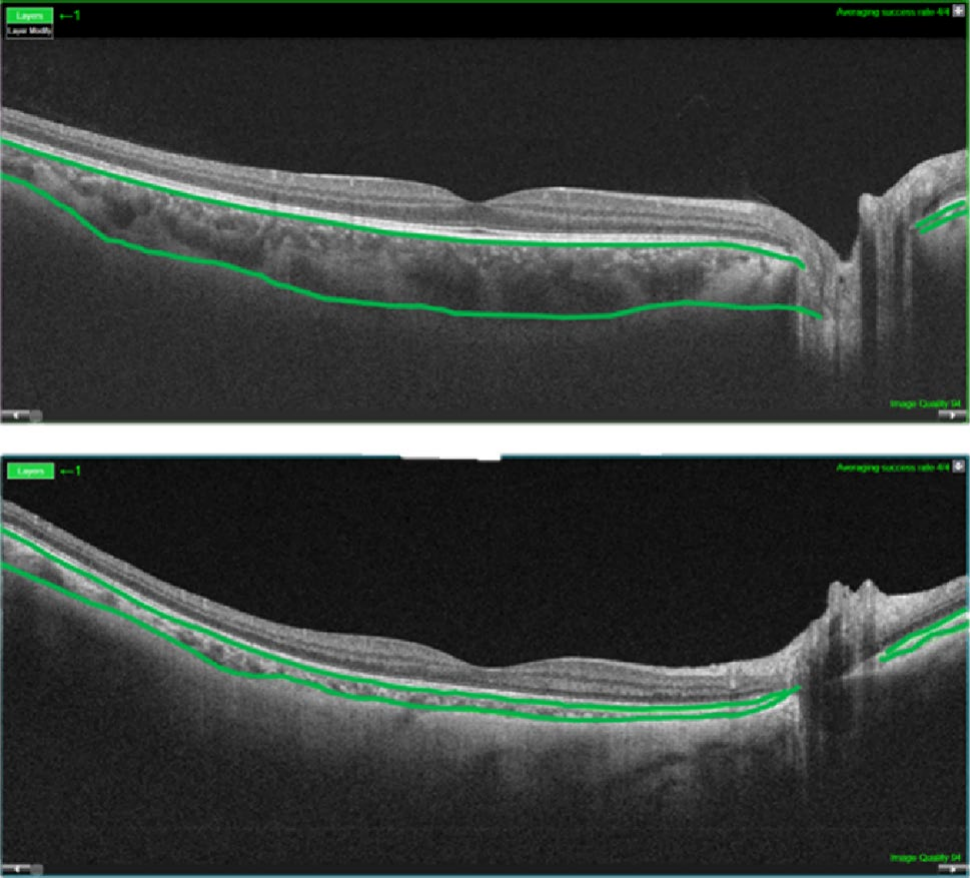
Differences in choroidal thickness between a patient with diffuse cutaneous SSc (upper panel) and a limited cutaneous SSc patient (lower panel)

Regarding the ocular features, dcSSc patients had a significantly reduced CC perfusion compared to lcSSc patients. However, the mean choroidal thickness (CT) in dcSSc patients was nearly twice as thick as the CT in lcSSc (304 ± 57 vs 176 ± 79 µm, *p* < 0.001). A comparison is shown in Fig. 1.

When SSc patients were stratified according to other features (presence or absence of ILD, esophageal involvement, PAH, kidney failure, previous and/or active digital ulcers, Scl70 or anti-centromere autoantibody positivity), choroidal thickness resulted significantly higher in Scl70 + patients vs Scl70− individuals and in patients who had previous digital ulcers (both *p* < 0.05, Supplementary Tables 1–7). Conversely, patients with anti-centromere positivity exhibited a significantly reduced choroidal thickness compared with ACA negative patients. Interestingly, there was a direct correlation between choroidal thickness and disease duration (*r* = 0.67, *p* = 0.001) among dcSSc patients reinforcing the hypothesis of a progressive fibrotic process increasing in individuals with longstanding disease.

**Fig. 2:**
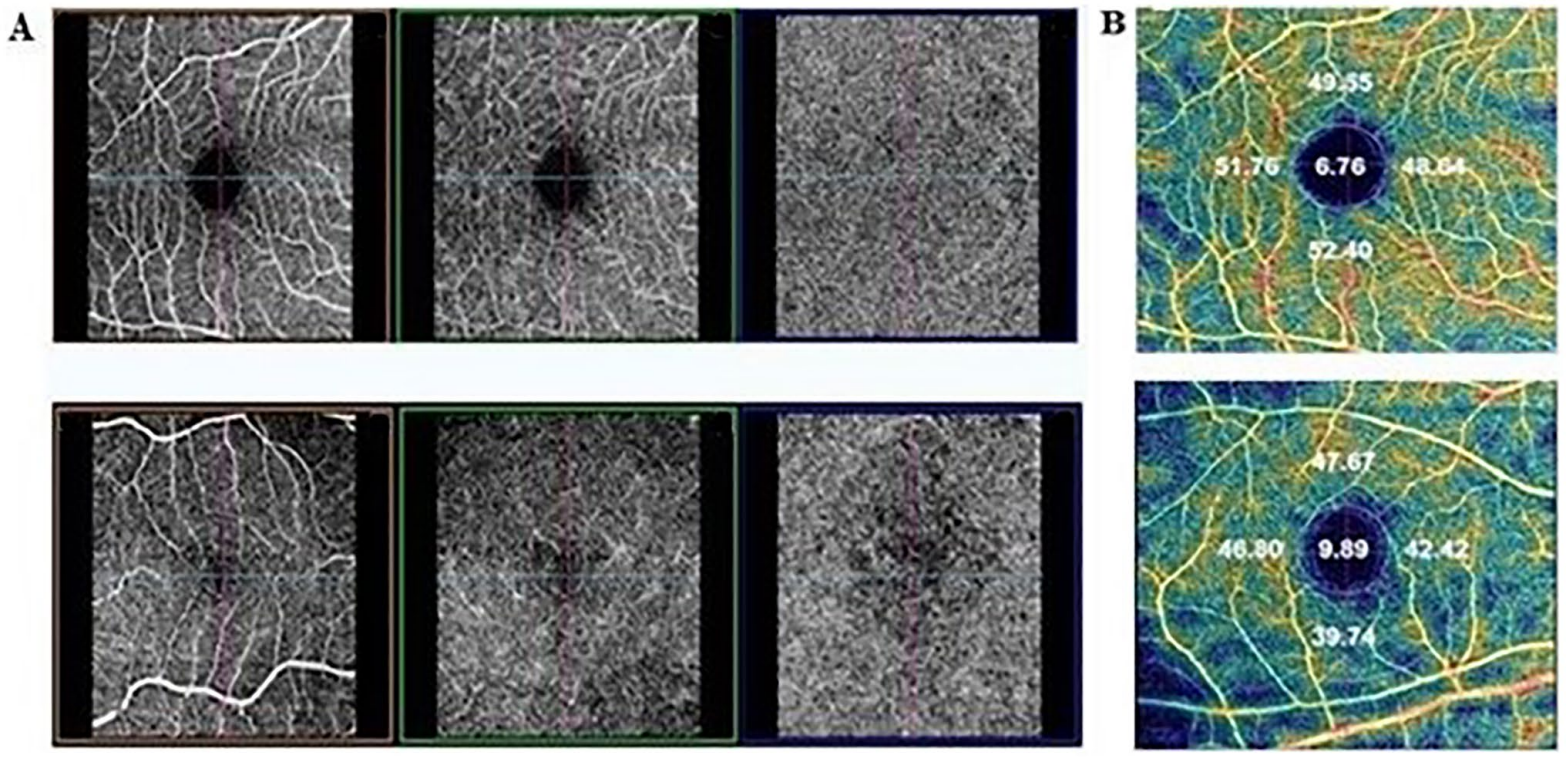
OCTA perfusion variables in SSc patients and healthy controls. **A** depicts the superficial vascular plexus (left column), deep vascular plexus (middle column), and choriocapillaris (right column) in a healthy control (upper row) and in a SSc patient (lower row), which is visibly reduced. **B** reports grids, graded according to the ETDRS protocol, showing the color-coded vessel density and perfusion in the superficial capillary plexus in a SSc patient (lower row) vs a healthy control (upper row). *ETDRS* early treatment diabetic retinopathy study

Table 3 shows OCT and OCTA measurements for both SSc and HCs. It is important to note that the structural features were similar, but there was a significant difference in all of the perfusion variables, including the superficial vascular plexus, deep vascular plexus, and choriocapillaris (Fig. 2).

To better investigate the association between the diagnosis of SSc and OCTA parameters, a logistic regression model was created (Supplementary Table 8). Notably, even after accounting for potential confounding factors such as age, axial length, scan quality, and intraocular pressure (IOP), the perfusion of the CC remained significant in predicting a diagnosis of SSc [OR = 0.66, 95% CI (0.45–0.96), *p* = 0.03].

For further analysis, we designed another model to assess the link between LASCA parameters—specifically, the mean value of perfusion units from the 2nd to the 5th finger—and SSc diagnosis, adjusting for age and gender (Supplementary Table 9). Analogously, this association also exhibited statistical significance [OR = 0.97, (0.96–0.99), *p* = 0.002].

Subsequently, a third model was created encompassing both OCTA and LASCA parameters. Even with the inclusion of both variables, the CC perfusion and the perfusion from the 2nd to the 5th finger at LASCA continued to demonstrate significant associations with SSc diagnosis (both *p* < 0.01, Supplementary Table 10).

To estimate the discriminatory capacity of these significant LASCA and OCTA variables in the distinction of SSc patients from HCs, we generated receiver operating characteristic (ROC) curves. Remarkably, when combining the performances of the OCTA parameters with the LASCA variables, the area under the curve (AUC) significantly increased compared to the individual ROC curves of OCTA and LASCA models (0.8 vs 0.76 vs 0.74, respectively) (Supplementary Fig. 1). In particular, the sensitivity and specificity of the combined model for the diagnosis of SSc were, respectively, estimated at 69% and 90% (see Supplementary Table 11 for the detailed cutoff values of each model). This enhancement in discriminatory ability reinforces the potential utility of using both OCTA and LASCA parameters together as valuable indicators for identifying SSc patients.

In the context of discerning between patients with dcSSc and lcSSc, ROC curves were employed to assess the discriminatory capacity of several parameters. Notably, only the measurement of the CT exhibited a noteworthy AUC value of 0.84, as shown in Supplementary Fig. 2. To elucidate further, a CT threshold of 211 μm was identified as the optimal point of demarcation, yielding sensitivity and specificity values of 85% and 69%, respectively, in the differentiation of dcSSc from lcSSc.

## Discussion

Our investigation showed that peripheral morphological and functional microvascular status in SSc patients, assessed by NVC and LASCA, significantly correlates with the impairment of ocular microcirculation.

More specifically, the mean capillary number at NVC directly correlated with the perfusion values of retinal vascular plexuses whereas the mean peripheral perfusion, detected by LASCA, positively correlated with both the retinal and choroidal perfusion.

When compared to healthy controls, a significant reduced percentage of perfusion was detected in both the superficial and deep retinal capillary plexus and in the choriocapillaris of SSc patients.

In light of this finding, OCTA might be conceivable as an emerging and viable modality for evaluating the systemic microangiopathy in SSc.

Indeed, in a recent study, NVC findings of SSc patients have shown to correlate with the choroidal vascular density and a negative correlation has been observed between the skin score and the vascular density of the superficial capillary plexus [11]. These capillaroscopic findings were observed in our SSc cohort for the retinal microcirculation and with the additional contribution of LASCA functional data reporting the correlations with the peripheral microvascular perfusion. Other reports have detected also correlations not only between chorioretinal microvasculature and NVC findings but also associations with pulmonary functions tests, particularly between perfusion of the CC and the diffusing capacity of the lungs for carbon monoxide (DLCO) [12]. In our subgroup analysis, we did not find significant differences of OCTA parameters between patients with or without ILD. This might be due to both the relatively small sample size of the subgroups and to the different definition of lung involvement which was defined by the radiologist based on HRTC assessment.

Other retinal features in SSc have been previously characterized by Ushiyama et al. [9] with an estimated prevalence of retinal disease (exudates, microhemorrhages, and macular degeneration) in 34% of SSc patients vs 8% of controls. Our cohort of SSc patients did not show these retinal findings, probably due to differences in the vasoactive and immunosuppressive treatment. Nevertheless, the diminished perfusion of the retinal and choroidal vascular structures observed in SSc patients through OCTA is in line with the findings reported in recent publications featuring smaller cohorts of SSc patients [26, 27].

In addition, observational studies and a recent systematic literature review reported that the choroidal vasculature might be more affected compared to the retinal microcirculation [5, 28].

In terms of other ocular features, SSc patients displayed a significantly higher IOP compared with HCs and the intraocular pressure values were inversely correlated with the mean number of capillaries per linear mm at NVC and the mean peripheral perfusion at LASCA from the 2nd to the 5th fingertip (*p* < 0.01).

These findings are consistent with literature reports reporting an increase in the IOP of SSc patients [29]. Mean IOP values in SSc patients have been simultaneously associated with higher corneal resistance factor suggesting a link with corneal biomechanical properties [30]. Interestingly, a cross-sectional study described glaucomatous abnormalities with a normal IOP which were detected in up to 20% of SSc patients [31]. These results were interpreted as related to vascular pathogenetic changes of normotensive glaucoma with potential perfusion abnormalities in the optic nerve head [31].

However, our findings indicated that, although there was a significant difference in IOP between SSc patients and HCs, the RNFL thickness was similar suggesting the presence of potential glaucomatous abnormalities in SSc individuals which are prevalently sub-clinical.

In our cohort, the average CT did not differ among SSc and HCs. However, we noticed that the mean CT was twice as high in dcSSc patients than in lcSSc patients despite a lower percentage of perfusion in the CC. This might be related to attempts of ocular microvascular reactivity and deposition of sub-endothelial extracellular matrix by resident choroidal fibroblasts in agreement with the fibrotic process observed in other tissues in such patients. As a matter of fact, patients with a higher disease duration displayed increased values of CT, and the other factors associated with this increase of thickness were the positivity for Scl70 and previous digital ulcers. Of note, morphological alterations of the choroidal vasculature, investigated by histology, have been previously reported by a post-mortem study of a patient with dcSSc highlighting a gross thickening of the internal elastic lamina [32]. Indeed, contrary to the retinal microcirculation considered an immunological sanctuary for the absence of resident fibroblasts and adrenergic vasomotor nerve supply, choroidal microvasculature is endowed with local fibroblasts which might be hyper-activated in pathological processes [33, 34].

Our analyses demonstrated also good discriminatory properties in distinguishing SSc patients from controls, particularly through the combined assessment of the mean perfusion from the 2nd to 5th finger at LASCA with choriocapillaris perfusion values, exhibiting a favorable AUC.

These results underscore the potentiality of these imaging techniques as valuable complementary diagnostic tools for SSc. Indeed, building on this, different recent studies are aiming to identify new complementary methods to enable the early diagnosis of SSc patients [35–38].

Among the main limitations of this study, we acknowledge the small sample size of SSc subgroups of patients and the difficulties in stratifying the differences of the OCTA variables among the “early”, “active” and “late” NVC patterns in SSc patients. This differentiation would have been potentially impaired also because of the low number of recruited “early” NVC pattern of SSc patients. Furthermore, our study design was cross-sectional, which limits our ability to infer causality between observed associations.

Nevertheless, this is the first study correlating both peripheral morphological and functional microvascular status with ocular findings in a SSc population with a reasonable sample size and free from cardiometabolic comorbidities which might have been a bias for assessing ocular and peripheral microcirculation. The strengths of our study design lie in the comprehensive assessment of both peripheral and ocular microvascular status, providing a holistic view of the microangiopathy in SSc patients, and in many subgroup analyses considering different features of the disease.

However, further investigations should explore the role of adding OCTA to standard exams to phenotypically stratify organ damage in SSc patients with the inclusion of ocular involvement, especially in evaluating the local microvascular damage and disease progression at follow-up [39]. Expanding sample size might allow for more detailed subgroup analyses according to the patient’s organ involvement and disease phenotype. Additional research avenues should be explored, including the exploration of early detection methodologies and their influence on the microvascular conditions in both peripheral and ocular contexts, as well as the exploration of associations with quality-of-life outcomes and comparative analyses against other autoimmune disorders.

## Conclusion

The altered morphological and functional peripheral microvascular status assessed at nailfold correlates with alterations in retinal and choriocapillaris microvasculature in SSc patients.

The ocular microvascular damage might explain both the increase of the intraocular pressure in SSc patients and the reduced perfusion of the posterior segment of the eye, similarly to the damage observed peripherally in the nailfold capillaries.

The increased thickness of the choroid, limited to the diffuse cutaneous forms of SSc and more frequent in patients with longstanding disease, Scl70 positivity and a previous history of digital ulcers, might be related to intensive ocular microvascular reactivity with progressive fibrosis, as observed in several tissues in such patients.

The synergistic utilization of the fingertip perfusion parameters, measured by LASCA, with the choroidal perfusion values at OCTA, might represent a compelling prospect as an adjunctive diagnostic support for the evaluation of the whole microcirculation in SSc patients.

### Supplementary Information

Below is the link to the electronic supplementary material.

Supplementary file1 (DOCX 397 KB)

## Data Availability

Data are available upon reasonable request.
