# Supplementary material for: Peripheral and ocular microvascular alterations in systemic sclerosis: observations from capillaroscopic assessments, perfusion peripheral analysis, and optical coherence tomography angiography
Source: Rheumatol Int. 2023 Nov 17;44(1):107–18. doi: 10.1007/s00296-023-05495-z (PMC10766778; doi:10.1007/s00296-023-05495-z)
Supplement: Supplementary file 1 — Supplementary file1 (DOCX 397 KB) [file 296_2023_5495_MOESM1_ESM.docx]

**Supplementary Figure 1.** ROC curves of mean peripheral perfusion detected by LASCA, OCTA parameters and combined variables.

**
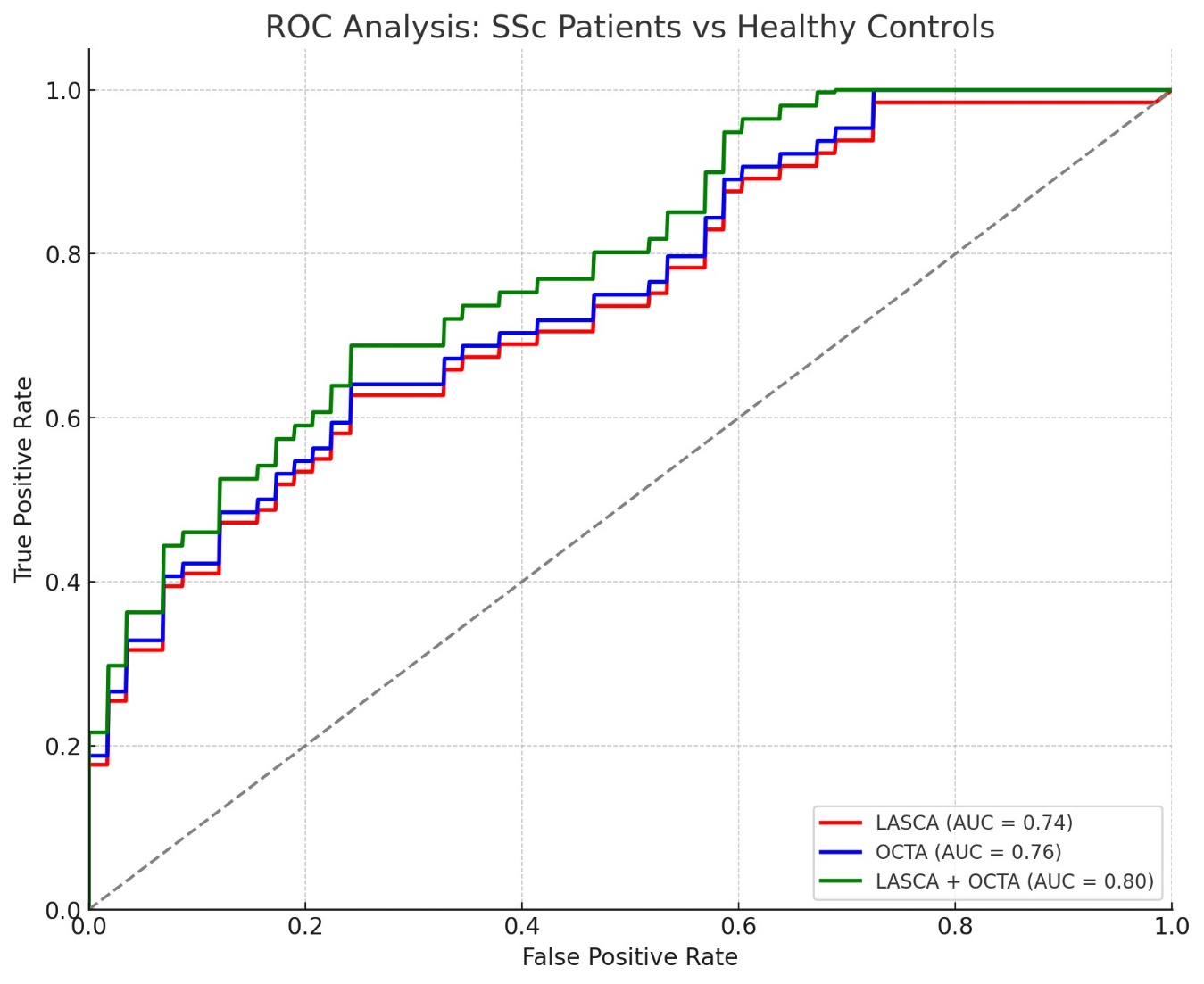
**

**Legend.** The red curve represents LASCA performance, the blue curve displays the performance of OCTA parameters (as combined assessments of SCP, DCP and CC) and the green curve is the combination of both. **Abbreviations**: LASCA, laser speckled contrast analysis; OCTA, optical coherence tomography angiography, AUC, area under the curve; SCP, superficial capillary plexus; DCP, deep capillary plexus; CC, choriocapillaris

**Supplementary Figure 2.** ROC curves of mean peripheral perfusion detected by LASCA, OCTA parameters, combined variables and choroidal thickness.


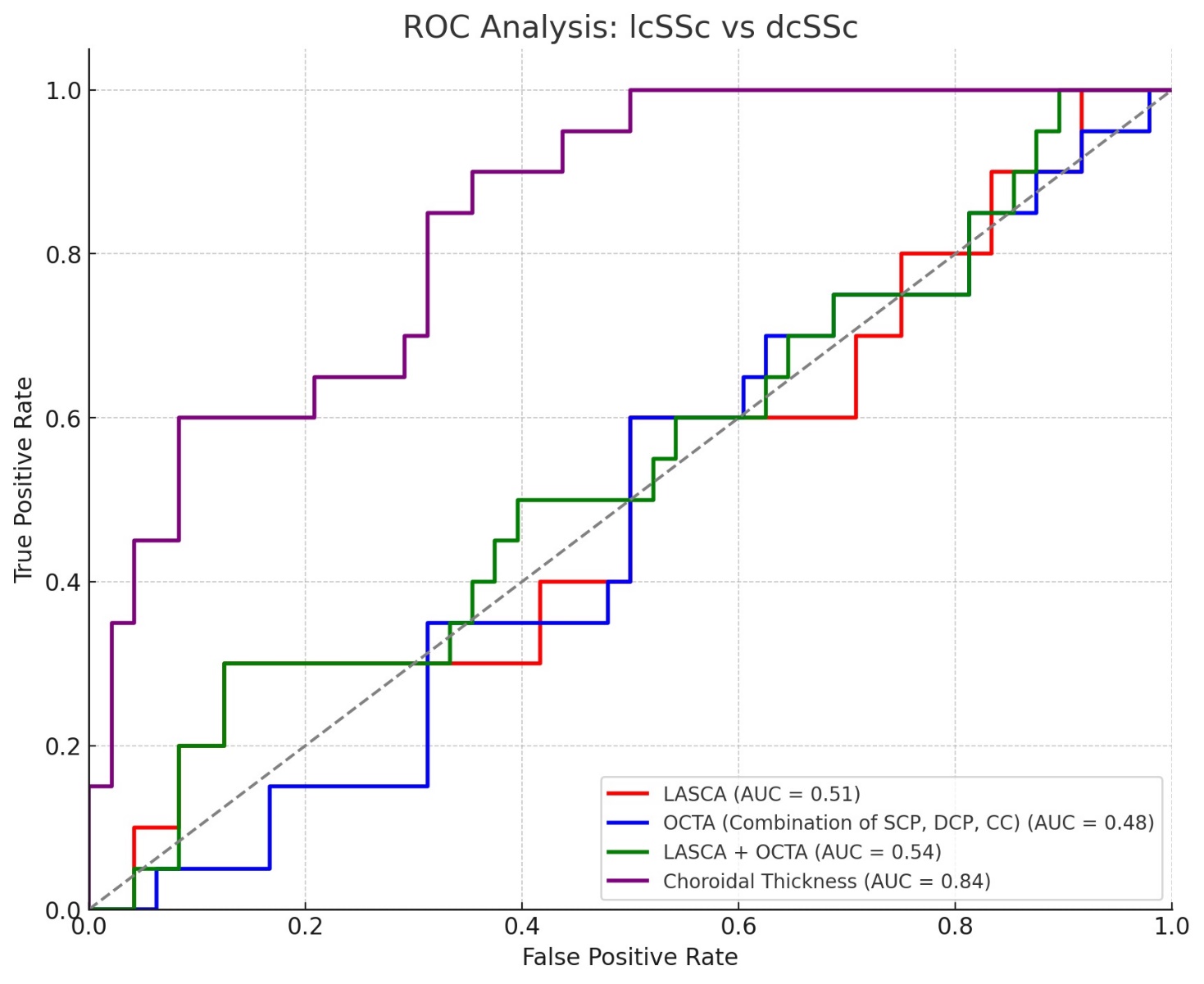


**Legend**. The red curve represents LASCA performance, the blue curve displays the performance of OCTA parameters (as combined assessments of SCP, DCP and CC), the green curve is the combination of both whereas the purple curve depicts the performance of the choroidal thickness. **Abbreviations**: LASCA, laser speckled contrast analysis, OCTA, optical coherence tomography angiography, AUC, area under the curve

**Supplementary Table 1.** Comparisons of OCTA parameters in SSc patients with and without ILD.

| **Parameter** | **mean ± SD**  **(patients with ILD, n = 20)** | **mean ± SD**  **(patients without ILD, n = 12)** | **p-value** |
| --- | --- | --- | --- |
| Superficial capillary plexus, % | 39.1 ± 3.1 | 37.5 ± 8.3 | 0.2669 |
| Deep capillary plexus, % | 39.2 ± 3.2 | 38.8 ± 8.2 | 0.5626 |
| Choriocapillaris, % | 50.8 ± 3.5 | 49.6 ± 10.2 | 0.5001 |
| Choroidal thickness, µm | 212.7 ± 88.6 | 192.2 ± 106.5 | 0.3844 |
| Retinal thickness, µm | 264.4 ± 33.8 | 265.9 ± 16.5 | 0.8281 |
| Retinal nerve fiber layer, µm | 101.5 ± 12.1 | 98.5 ± 13.3 | 0.3379 |

**Abbreviations.** SD, standard deviation; ILD, interstitial lung disease, %, percentage of perfusion.

ILD was dichotomously defined by a radiologist on the high-resolution computerized tomography (HRCT) findings.

**Supplementary Table 2.** Comparisons of OCTA parameters in SSc patients with and without oesophageal involvement.

| **Parameter** | **mean ± SD**  **(patients with oesophageal involvement, n = 14)** | **mean ± SD**  **(patients without oesophageal involvement, n = 18)** | **p-value** |
| --- | --- | --- | --- |
| Superficial capillary plexus, % | 38.9 ± 2.4 | 37.4 ± 7.2 | 0.0907 |
| Deep capillary plexus, % | 39.6± 3.1 | 38.3 ± 7.1 | 0.3463 |
| Choriocapillaris, % | 50.5 ± 3.5 | 50.3 ± 8.7 | 0.9076 |
| Choroidal thickness, µm | 226.2 ± 97.7 | 188.7 ± 87.3 | 0.1063 |
| Retinal thickness, µm | 271.9 ± 13.3 | 259.9 ± 34.6 | 0.0843 |
| Retinal nerve fiber layer, µm | 103.2 ± 9.8 | 98.2 ± 14.1 | 0.1145 |

**Abbreviations.**  SD, standard deviation; %, percentage of perfusion.

Oesophageal involvement was defined if motor alterations were detected by a gastroenterologist at pH-manometry.

**Supplementary Table 3.** Comparisons of OCTA parameters in SSc patients with and without PAH.

| **Parameter** | **mean ± SD**  **(patients with pulmonary arterial hypertension, n = 5)** | **mean ± SD**  **(patients without pulmonary arterial hypertension, n = 27)** | **p-value** |
| --- | --- | --- | --- |
| Superficial capillary plexus, % | 39.1 ± 1.8 | 38.4 ± 6.3 | 0.7422 |
| Deep capillary plexus, % | 40.3 ± 2.1 | 38.6 ± 6.1 | 0.3967 |
| Choriocapillaris, % | 51.7 ± 1.6 | 50.1 ± 7.5 | 0.5168 |
| Choroidal thickness, µm | 175.3 ± 124.8 | 209.9 ± 86.6 | 0.2822 |
| Retinal thickness, µm | 265.8 ± 5.9 | 264.8 ± 30.5 | 0.9216 |
| Retinal nerve fiber layer, µm | 99.1 ± 10.8 | 100.5 ± 12.9 | 0.7441 |

**Abbreviations.** SD, standard deviation; PAH, pulmonary arterial hypertension, %, percentage of perfusion.

PAH was defined either dichotomously as unequivocable echocardiographic changes combined with other clinical features (pulmonary artery systolic pressure > 40 mmHg combined with changes of the right ventricle [RV] such as RV enlargement, RV hypertrophy, flattened interventricular septum, significant unexplained dyspnoea disproportionate to lung involvement and DLCO reduction at pulmonary function tests) or as a mean PAH > 20 mmHg at right heart catheterization (RHC).

Only in 3/5 patients the measurements were obtained with RHC. In the other two cases, only echocardiogram was performed due to denied consent of one patient to perform the procedure and in the other case was postponed due a high risk of peri-procedural complications for the other patient at the moment of evaluation (renal insufficiency and pneumonia).

**Supplementary Table 4.** Comparisons of OCTA parameters in SSc patients with and without kidney involvement.

| **Parameter** | **mean ± SD**  **(patients with kidney involvement, n = 11)** | **mean ± SD**  **(patients without kidney involvement, n = 21)** | **p-value** |
| --- | --- | --- | --- |
| Superficial capillary plexus, % | 38.7 ± 3.2 | 39.7 ± 2.4 | 0.2124 |
| Deep capillary plexus, % | 36.9 ± 8.9 | 39.8 ± 2.9 | 0.0793 |
| Choriocapillaris, % | 48.9 ± 11.5 | 51 ± 3.1 | 0.2649 |
| Choroidal thickness, µm | 224.1 ± 109.7 | 196.2 ± 84.8 | 0.2672 |
| Retinal thickness, µm | 266.7 ± 16.4 | 262.2 ± 32.1 | 0.7486 |
| Retinal nerve fiber layer, µm | 99.1 ± 12.9 | 102.5 ± 11.9 | 0.0947 |

**Abbreviations.**  SD, standard deviation; %, percentage of perfusion.

Kidney involvement was defined if there was laboratory evidence of renal failure (serum creatinine > 1.2 mg/dL and an estimated glomerular filtration rate < 60 ml/min/1.73m^2^) or as increased resistive indexes of renal arteries at vascular doppler ultrasonography.

**Supplementary Table 5.** Comparisons of OCTA parameters in SSc patients with and without previous digital ulcers*.

| **Parameter** | **mean ± SD**  **(patients with previous digital ulcers, n = 13)** | **mean ± SD**  **(patients without a history of digital ulcers, n = 19)** | **p-value** |
| --- | --- | --- | --- |
| Superficial capillary plexus, % | 39.4 ± 2.9 | 37.9 ± 7.1 | 0.3119 |
| Deep capillary plexus, % | 40.3 ± 2.9 | 37.9 ± 6.8 | 0.1007 |
| Choriocapillaris, % | 50.7 ± 3.1 | 50.1 ± 8.6 | 0.7228 |
| **Choroidal thickness, µm** | **234.6 ± 93.1** | **185.2 ± 88.8** | **0.0341** |
| Retinal thickness, µm | 272.3 ± 15.1 | 260.2 ± 33.3 | 0.0857 |
| Retinal nerve fiber layer, µm | 102.5 ± 12.7 | 98.9 ± 12.5 | 0.2545 |

**Abbreviations.**  SD, standard deviation; %, percentage of perfusion.

* a subgroup analysis for patients with *active* digital ulcers was not performed due to the presence of only one patient having active digital ulcers.

**Supplementary Table 6.** Comparisons of OCTA parameters in SSc patients with and without Scl70 positivity.

| **Parameter** | **mean ± SD**  **(patients with Scl70 positivity, n = 10)** | **mean ± SD**  **(patients with negative Scl70, n = 22)** | **p-value** |
| --- | --- | --- | --- |
| Superficial capillary plexus, % | 38.5 ± 3.3 | 38.4 ± 6.6 | 0.9439 |
| Deep capillary plexus, % | 39.5 ± 3.3 | 38.6 ± 6.5 | 0.5157 |
| Choriocapillaris, % | 50.1 ± 3.6 | 50.5 ± 8.1 | 0.8524 |
| Choroidal thickness, µm | **258.4 ± 91.2** | **181.3 ± 84.6** | **0.0015** |
| Retinal thickness, µm | 275.4 ± 14.3 | 267.4 ± 31.4 | 0.1461 |
| Retinal nerve fiber layer, µm | 100.5 ± 13.6 | 100.2 ± 12.3 | 0.9342 |

**Abbreviations.** SD, standard deviation; Scl70, anti-topoisomerase I antibody; %, percentage of perfusion

**Supplementary Table 7.** Comparisons of OCTA parameters in SSc patients with and without anti-CENPA/B positivity.

| **Parameter** | **mean ± SD**  **(patients with anti-CENP A/B positivity, n = 10)** | **mean ± SD**  **(patients with negative anti-CENP A/B, n = 22)** | **p-value** |
| --- | --- | --- | --- |
| Superficial capillary plexus, % | 38.3 ± 8 | 38.5 ± 3.8 | 0.8797 |
| Deep capillary plexus, % | 38.2 ± 8.3 | 39.3 ± 3 | 0.4344 |
| Choriocapillaris, % | 49.6 ± 10.4 | 50.9 ± 2.9 | 0.4591 |
| **Choroidal thickness, µm** | **175.4 ± 89.9** | **224.8 ± 90.9** | **0.0329** |
| Retinal thickness, µm | 266.2 ± 37.9 | 271.1 ± 16.5 | 0.1326 |
| Retinal nerve fiber layer, µm | 98.4 ± 11.7 | 101.6 ± 13.2 | 0.3169 |

**Abbreviations.** SD, standard deviation; %, percentage of perfusion

**Supplementary Table 8.** OCTA characteristics associated with SSc

|  | **Univariate** | | | **Multivariate** | | |
| --- | --- | --- | --- | --- | --- | --- |
|  | **OR** | **95% C.I.** | **p** | **OR** | **95% C.I.** | **p** |
| Age, years | 1.01 | 0.97 to 1.05 | 0.477 | 0.99 | .94 to 1.06 | 0.954 |
| Gender, female | 0.43 | 0.77 to 2.43 | 0.341 | 0.60 | 0.76 to 4.55 | 0.612 |
| Axial length | 1.16 | 0.78 to 1.72 | 0.463 | 1.11 | 0.59 to 2.07 | 0.745 |
| Scan quality | 0.96 | 0.87 to 1.05 | 0.368 | 0.99 | 0.87 to 1.13 | 0.901 |
| Superficial vascular plexus, % | 0. 79 | 0.63 to 0.99 | 0.048 |  |  |  |
| Deep vascular plexus, % | 0.79 | 0.64 to 0.98 | 0.035 |  |  |  |
| Choriocapillaris, % | 0.57 | 0.38 to 0.87 | 0.008 | 0.66 | 0.45 to .96 | **0.030** |
| Choroidal thickness, µm | 0.99 | 0.99 to 1.01 | 0.720 | 0.999 | .99 to 1.01 | 0.861 |
| Retinal thickness, µm | 0.98 | 0.94 to 1.01 | 0.207 |  |  |  |
| RNFL, µm | 0.990 | 0.942 to 1.040 | 0.702 |  |  |  |
| IOP, mmHg | 1.42 | 1.10 to 1.84 | 0.008 | 1.31 | 1.00 to 1.74 | 0.058 |

**Legend.** RNFL: retinal nerve fiber layer; IOP: intraocular pressure. Model 1. Multivariate model: p=0.015, pseudo R2=0.198.

**Supplementary Table 9.** LASCA parameters associated with SSc.

|  | Univariate | | | Multivariate | | |
| --- | --- | --- | --- | --- | --- | --- |
|  | OR | 95% C.I. | p | OR | 95% C.I. | p |
| Age, years | 1.01 | 0.97 to 1.05 | 0.477 | 1.03 | 0.97 to 1.08 | 0.268 |
| Gender, female | 0.43 | 0.77 to 2.43 | 0.341 | 0.87 | 0.12 to 6.13 | 0.889 |
| LASCA perfusion from 2^nd^ to 5^th^ finger | 0.98 | .97 to .99 | 0.002 | .9841 | .97406 to .994250 | **0.002** |

Model 2 Multivariate model: p<0.001, pseudo R2= 0.173.

**Supplementary Table 10.** Ocular and LASCA features associated with SSc.

|  | **Multivariate** | | |
| --- | --- | --- | --- |
|  | **OR** | **95% C.I.** | **p** |
| Age, years | 1.02 | .99 to 1.05 | 0.280 |
| Gender, female | 0.74 | 0.23 to 2.37 | 0.611 |
| LASCA perfusion from 2^nd^ to 5^th^ finger | 0.98 | 0.98 to 0.99 | **<0.01** |
| Choriocapillaris, % | 0.77 | 0.65 to 0.91 | **0.003** |
| IOP, mmHg | 1.22 | 1.04 to 1.44 | 0.13 |

Model 3. Multivariate model: p<0.001, AUC= 0.247.

**Supplementary Table 11**. Performance of OCTA or LASCA incorporated models in the prediction of SSc.

| **Variable** | **Sensitivity (95% C.I.) *** | **Specificity (95% C.I.) *** | **Area under the curve (95% C.I.)** |
| --- | --- | --- | --- |
| Superficial vascular plexus, % | 0.75 (0.54 0.95) | 0.54 (0.29 0.79) |  |
| Deep vascular plexus, % | 0.63 (0.45 0.81) | 0.70 (0.53 0.88) |  |
| Choriocapillaris, % | 0.58 (0.47 0.69) | 0.80 (0.71 0.90) |  |
| LASCA perfusion from 2^nd^ to 5^th^ finger, PU | 0.77 (0.62 0.93) | 0.70 (0.57 0.83) |  |
| Multivariate models |  |  |  |
| 1 | 0.72 (0.48 0.93) | 0.72 (0.49 0.94) | 0.76 (0.69 0.83) |
| 2 | 0.77 (0.69 0.86) | 0.81 (0.72 0.91) | 0.74 (0.67 0.82) |
| 3 | 0.69 (0.49 0.88) | 0.90 (0.71 1.09) | 0.80 (0.74 0.87) |
